# Supplementary material for: Metabolomics of sebum reveals lipid dysregulation in Parkinson’s disease
Source: Nat Commun. 2021 Mar 11;12:1592. doi: 10.1038/s41467-021-21669-4 (PMC7952564; doi:10.1038/s41467-021-21669-4)
Supplement: Supplementary file 1 — Supplementary Information [file 41467_2021_21669_MOESM1_ESM.pdf]

Metabolomics of sebum reveals lipid dysregulation in Parkinson's disease

Eleanor Sinclair[1], Drupad K Trivedi[1], Depanjan Sarkar[1], Caitlin Walton-Doyle[1], Joy Milne[1], Tilo Kunath[2], Anouk M. Rijs[3], Rob M. A. de Bie[4], Royston Goodacre[5] Monty Silverdale[6] and Perdita Barran*[1]

[1]Manchester Institute of Biotechnology, School of Chemistry, The University of Manchester, Princess Street, Manchester, UK, M1 7DN
[2]Institute for Stem Cell Research, School of Biological Sciences, The University of Edinburgh, Edinburgh UK, EH16 4UU
[3]Division of BioAnalytical Chemistry, AIMMS Amsterdam Institute of Molecular and Life Sciences, Vrije Universiteit Amsterdam, De Boelelaan 1108, 1081 HZ Amsterdam, The Netherlands
[4]Amsterdam University Medical Centers, University of Amsterdam, Department of Neurology, Amsterdam Neuroscience, Meibergdreef 9, 1105 AZ Amsterdam, The Netherlands
[5]Institute of Systems, Molecular and Integrative Biology, Department of Biochemistry and Systems Biology, University of Liverpool, Crown Street, Liverpool L69 7ZB
[6]Department of Neurology, Salford Royal Foundation Trust, Manchester Academic Health Science Centre, University of Manchester, UK

Supplementary Information

Table of Contents

Table S1. Statistical significance between drug naïve PD and control cohorts and medicated PD and control cohorts 3

Table S2. PLS-DA classification results for gender separated models of drug naïve PD *vs.* control and medicated PD *vs.* control 4

Figure S1. PLS-DA classification model output for medicated PD vs. drug naïve PD, when medicated PD is the 'positive' predictive class, ... 5

Figure S2. Bar charts displaying the area under the curve (AUC) from univariate ROC analysis 6

Table S3A. Putative annotations supported by MS/MS fragmentation data of VIP compounds common to both drug naïve PD vs. control and medicated PD vs. control analyses (VIP > 1). 7

Table S3B. A list of the database matches based upon accurate mass (Lipid Maps and METLIN, 10 ppm) for VIP compounds common to both drug naïve PD *vs.* control and medicated PD *vs.* control analyses (VIP > 1). 7

Table S4A. Putative annotations supported by MS/MS fragmentation data for VIP compounds found only in drug naïve PD *vs.* control analysis (VIP > 1). 9

Table S4B. A list of the database matches based upon accurate mass (Lipid Maps and METLIN, 10 ppm) for VIP compounds found only in drug naïve PD *vs.* control analysis (VIP > 1). 9

Table S5A. Putative annotations supported by MS/MS fragmentation data of VIP compounds found only in medicated PD *vs.* control analysis (VIP > 1). 11

Table S5B. A list of the database matches based upon accurate mass (Lipid Maps and METLIN, 10 ppm) for VIP compounds found only in drug naïve PD *vs.* control analysis (VIP > 1). 12

Figure S3. Pearson's correlation matrix displaying the relationship between the number of alcohol units consumed per participant and the intensity of each corresponding VIP compound from PLS-DA modelling. 14

Table S6. Mummichog output from MetaboAnalyst analysis for pathways with $p < 0.05$ in drug naïve PD *vs.* control cohorts 15

Table S7. Mummichog output from MetaboAnalyst analysis for pathways with $p < 0.05$ in medicated PD *vs.* control cohorts 16

Table S8. Details of the collecting sites in the UK/NL and the lead PI at each site 17

Table S1. Statistical significance between drug naïve PD and control cohorts and medicated PD and control cohorts

	Drug Naïve PD - Control	Significant	Medicated PD - Control	Significant
Age[a]	5.73×10^{-11}	Yes	6.25×10^{-14}	Yes
BMI (kg/m^2)[a]	0.640	No	0.880	No
Gender[b]	0.045	Yes	0.042	Yes
Alcohol Intake[b]	0.192	No	0.015	Yes
Smoker[b,c]	0.837	No	0.006	Yes

[a] Mann Whitney non-parametric U test (two-tailed) used to determine significance, *p*-values were calculated at the 0.05 confidence level
[b] Chi-squared test implemented for categorical variables
[c] Fisher's exact test employed to calculate significance of smokers *vs.* non-smokers within medicated PD and control groups

Table S2. PLS-DA classification results for gender separated models of drug naïve PD *vs.* control and medicated PD *vs.* control

PLS-DA classification rates	**Male** (*n*=163)		**Female** (*n*=111)	
	Drug Naïve PD *vs.* Control	Medicated PD *vs.* Control	Drug Naïve PD *vs.* Control	Medicated PD *vs.* Control
Averaged CCR	68.9%	60.2%	57.1%	70.2%
Sensitivity	56.4%	65.5%	63.5%	76.6%
Specificity	62.6%	41.7%	49.0%	52.3%

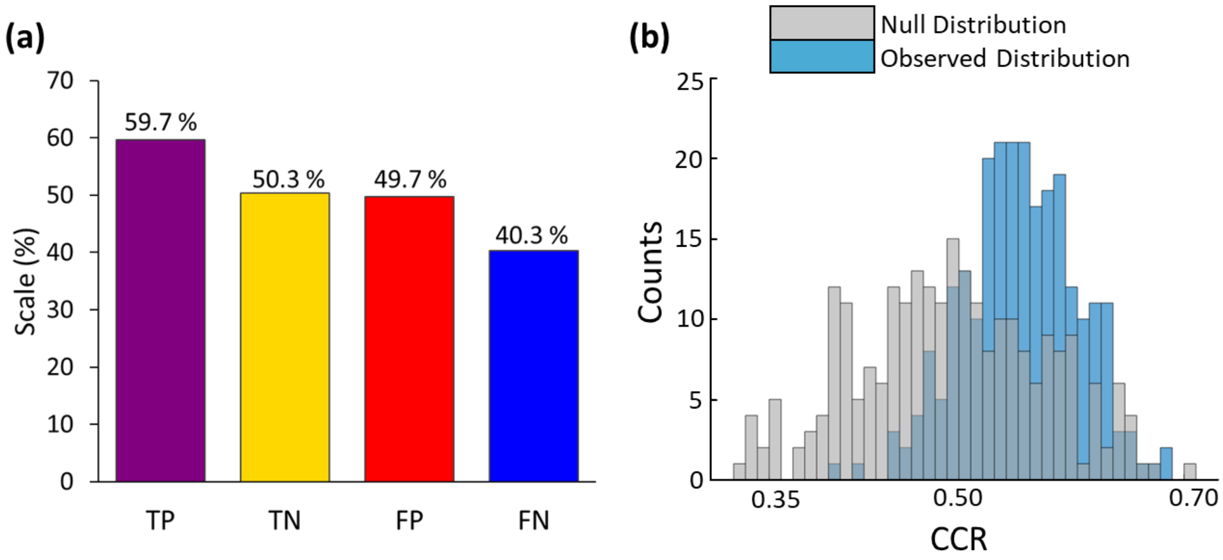

Figure S1. PLS-DA classification model output for medicated PD vs. drug naïve PD, when medicated PD is the 'positive' predictive class, (a) bar chart displaying the true positive, true negative, false positive and false negative rates from PLS-DA modelling, (b) frequency histogram to present the correct classification rate of bootstrap validation (*n*=250 resampling models), grey bars represent the null dristribution and blue bars signify the observed distribution.

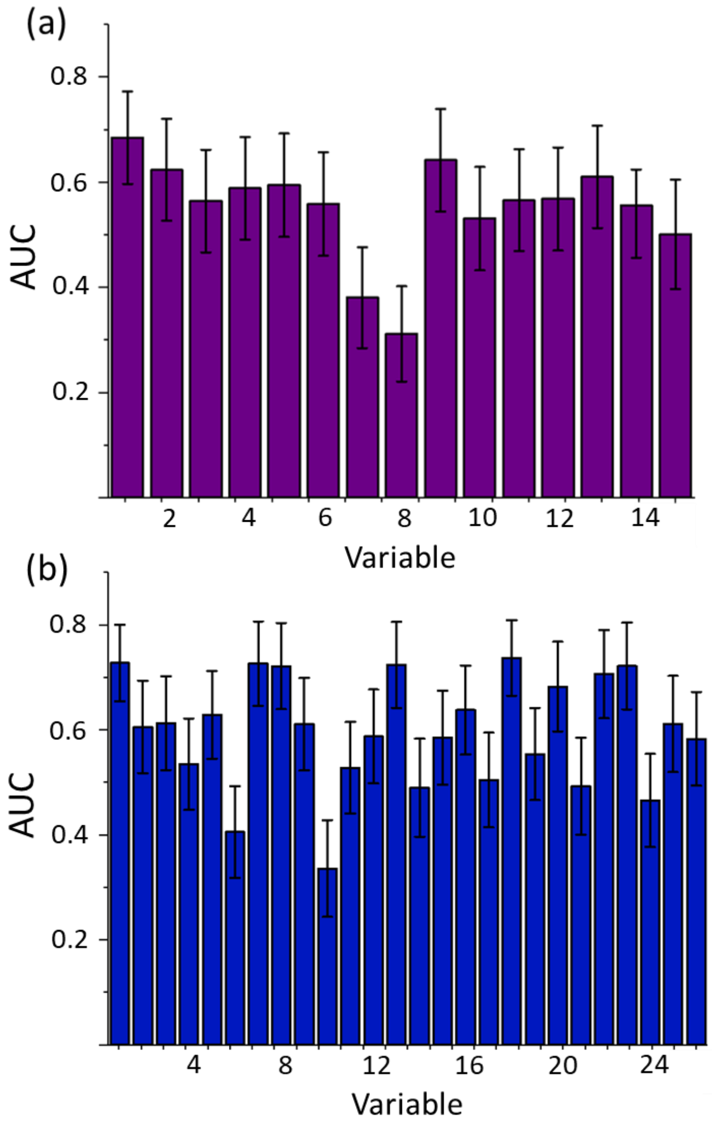

Figure S2. Bar charts displaying the area under the curve (AUC) from univariate ROC analysis for (a) drug naïve PD *vs.* control ROC analysis for variables with VIP > 1 (variables, *n*=15) using PLS-DA variable ranking, (b) medicated PD *vs.* control analysis ROC analysis for variables with VIP >1 (variables, *n*=26) using PLS-DA variable ranking. Data are presented as mean AUC value with error bars representing the minima and maxima values of the 95% CI range

Variables correspond to VIP features as follows:

(a) #1. *m/z* 368.4242, #2. *m/z* 550.6277, #3. *m/z* 666.6370, #4. *m/z* 638.6067, #4. *m/z* 256.2645, #5. *m/z* 522.5965, #6. *m/z* 668.6178, #7. *m/z* 764.568, #8. *m/z* 194.1396, #9. *m/z* 825.6939, #10. *m/z* 358.3677, #11. *m/z* 692.6168, #12. *m/z* 610.5763, 13. *m/z* 494.5656, #14. *m/z* 430.3881, #15. *m/z* 414.4308

(b) #1. *m/z* 368.4242, #2. *m/z* 666.6370, #3. *m/z* 638.6067, #4. *m/z* 256.2645, #5. *m/z* 550.6277, #6. *m/z* 764.5681, #7. *m/z* 830.7349, #8. *m/z* 825.6939, #9. *m/z* 610.5763 #10. *m/z* 194.1396, #11. *m/z* 283.2885, #12. *m/z* 338.3426, #13. *m/z* 839.7100, #14. *m/z* 641.4795, #15. *m/z* 664.6213, #16. *m/z* 402.3932, #17. *m/z* 894/6996, #18. *m/z* 340.3935**,** #19. *m/z* 358.3677, #20. *m/z* 369.3836**,** #21. *m/z* 553.43, #22. *m/z* 827.7101, #23. *m/z* 788.6888n, #24. *m/z* 792.5955, #25. *m/z* 414.4308, #26. *m/z* 636.5911

Table S3A. Putative annotations supported by MS/MS fragmentation data of VIP compounds common to both drug naïve PD vs. control and medicated PD vs. control analyses (VIP > 1).

Putative Annotation	Formula	Adduct	Score	Fragmentation Score	Isotope Similarity	Neutral Mass	*m/z*	Δ ppm	Expression Drug Naïve PD (Fold Change)	Expression Medicated PD (Fold Change)
TG(50:5)	$C_{53}H_{92}O_6$	$[M+H]^+$	47.9	60.3	83.43	824.6894	825.6939	3.4	↓ (0.77)	↓ (0.64)
HexCer(36:2)	$C_{42}H_{79}NO_9$	$[M+Na]^+$	46.5	47.0	90.65	741.5755	764.5681	4.4	↑ (1.15)	↑ (1.10)
Cer(42:0)	$C_{42}H_{85}NO_5$	$[M-H_2O+H]^+$	45.7	41.7	90.00	683.6428	666.6370	3.7	↓ (0.60)	↓ (0.47)
Cer(40:0)	$C_{40}H_{81}NO_5$	$[M-H_2O+H]^+$	46.0	37.5	92.83	655.6115	638.6067	2.3	↓ (0.61)	↓ (0.47)
Cer(38:1)	$C_{38}H_{75}NO_4$	$[M+H]^+$	49.6	50.3	97.66	609.5696	610.5763	1.0	↓ (0.63)	↓ (0.48)

TG (Triacylglycerol); HexCer (Hexosylceramide); Cer (Ceramide)
Fold Change = PD (Intensity) / Control (Intensity); where PD refers to Drug Naïve or Medicated cohorts, respectively

Table S3B. A list of the database matches based upon accurate mass (Lipid Maps and METLIN, 10 ppm) for VIP compounds common to both drug naïve PD *vs.* control and medicated PD *vs.* control analyses (VIP > 1).

Measured Feature	Database Matches	Formula	Adduct	Neutral Mass	Δ ppm	Expression Drug Naïve PD (Fold Change)	Expression Medicated PD (Fold Change)	Lipid Maps	METLIN
m/z 414.4308	FA(26:0)*	$C_{26}H_{52}O_2$	$[M+NH_4]^+$	396.3967	0.6	↑ (1.23)	↓ (0.84)	✓	✓
	Methyl pentacosanoate**								✓
m/z 358.3677	FA(22:0)*	$C_{22}H_{44}O_2$	$[M+NH_4]^+$	340.3341	0.7	↓ (0.81)	↓ (0.78)	✓	✓
m/z 194.1396	FA(8:0);O2*	$C_8H_{16}O_4$	$[M+NH_4]^+$	176.1049	4.7	↑ (1.74)	↑ (1.78)	✓	✓
	L-Cladinose								✓
	Metaldehyde†								✓

m/z 550.6277	-	-	-	-	-	↑ (1.33)	↑ (1.10)	-	
m/z 368.4242	-	-	-	-	-	↓ (0.15)	↓ (0.14)	-	

FA (Fatty Acyl)
Lipid ID * corresponds to a Lipid Maps Structure Database (LMSD) hit which comprises biologically relevant lipids to mammalian species. All other database matches correspond to Lipid Maps Computationally-generated species (COMP_DB) and METLIN as indicated in the final two columns
The number of oxygen atoms that are not included in the class-specific functional group is added after the semi-colon, where applicable
**Fatty acid methyl ester
†Pesticide
Fold Change = PD (Intensity) / Control (Intensity); where PD refers to Drug Naïve or Medicated cohorts, respectively

Table S4A. Putative annotations supported by MS/MS fragmentation data for VIP compounds found only in drug naïve PD *vs.* control analysis (VIP > 1).

Putative Annotation	Formula	Adduct	Score	Fragmentation Score	Isotope Similarity	Neutral Mass	*m/z*	Δ ppm	Expression Drug Naïve PD (Fold Change)
DG(38:1)	$C_{41}H_{78}O_5$	$[M+NH_4]^+$	48.6	50.4	95.69	650.5849	668.6178	1.4	↓ (0.81)

DG (Diacylglycerol)
Fold Change = Drug Naïve PD (Intensity) / Control (Intensity)

Table S4B. A list of the database matches based upon accurate mass (Lipid Maps and METLIN, 10 ppm) for VIP compounds found only in drug naïve PD *vs.* control analysis (VIP > 1).

Measured Feature	Database Matches	Formula	Adduct	Neutral Mass	Δ ppm	Expression Drug Naïve PD (Fold Change)	Lipid Maps	METLIN
m/z 430.3881	DG O(22:1)	$C_{25}H_{48}O_4$	$[M+NH_4]^+$	412.3547	2.3	↓ (0.75)	✓	
	FA(25:1);O2*						✓	
	MG(22:1)						✓	
	MG O(22:2);O						✓	✓
	NAE(23:0)	$C_{25}H_{51}NO_4$	$[M+H]^+$	429.3813	2.3		✓	
m/z 692.6168	Cer(41:0);O4*	$C_{41}H_{83}NO_5$	$[M+Na]^+$	669.6271	0.7	↓ (0.53)	✓	✓
	DG(40:3)*	$C_{43}H_{78}O_5$	$[M+NH_4]^+$	674.5849	2.8		✓	✓
	DG O(40:4);O						✓	
	TG O(40:3)						✓	
	Cer(43:3);O4	$C_{43}H_{81}NO_5$	$[M+H]^+$	667.6115	2.8		✓	
	Acer(43:2);O3						✓	

	Cer(43:2);O5<br>ACer(43:1);O4	$C_{43}H_{83}NO_6$	$[M-H_2O+H]^+$	709.6220	2.8		✓<br>✓	
m/z 522.5965	-	-	-	-	-	↓ (0.62)		
m/z 494.5656	-	-	-	-	-	↓ (0.45)		

DG O (Alkylacylglycerol); FA (Fatty Acyl); MG (Monoacylglycerol); MG O (Monoalkylglycerol); NAE (N-Acyl Ethanolamine); Cer (Ceramide); DG (diacylglyerol); TG O (Alkyldiacylglycerol); ACer (Acyl ceramide)

The number of oxygen atoms that are not included in the class-specific functional group is added after the semi-colon, where applicable

Lipid ID * corresponds to a Lipid Maps Structure Database (LMSD) hit which comprises only biologically relevant lipids to mammalian species. All other database matches correspond to Lipid Maps Computationally-generated species (COMP_DB) and METLIN as indicated in the final two columns

Fold Change = Drug Naïve PD (Intensity) / Control (Intensity)

Table S5A. Putative annotations supported by MS/MS fragmentation data of VIP compounds found only in medicated PD *vs.* control analysis (VIP > 1).

Putative Annotation	Formula	Adduct	Score	Fragmentation Score	Isotope Similarity	Neutral Mass	*m/z*	Δ ppm	Expression Medicated PD (Fold Change)
HexCer(44:1)	$C_{50}H_{97}NO_{10}$	$[M+Na]^+$	53.9	71.4	98.15	871.7112	894.6996	1.0	↓ (0.99)
TG(50:2)	$C_{53}H_{98}O_6$	$[M+Na]^+$	44.8	38.9	86.88	830.7363	853.7241	1.8	↓ (0.64)
TG(49:2)	$C_{52}H_{96}O_6$	$[M+Na]^+$	44.6	30.0	93.56	816.7207	839.7100	0.1	↓ (0.64)
TG(48:1)	$C_{51}H_{96}O_6$	$[M+Na]^+$	49.6	49.0	98.99	804.7207	827.7101	0.4	↓ (0.67)
TG(47:2)	$C_{50}H_{92}O_6$	$[M+Na]^+$	45.2	31.9	94.50	788.6894	811.6787	0.4	↓ (0.62)
HexCer(38:2)	$C_{44}H_{83}NO_9$	$[M+Na]^+$	48.8	48.6	95.31	769.6068	792.5955	6.7	↑ (1.03)
SPH(18:0)	$C_{18}H_{39}NO_2$	$[M-H_2O+H]^+$	46.0	33.5	99.21	301.2981	284.2958	3.4	↓ (0.94)
Hexadecadienol	$C_{16}H_{33}NO$	$[M+NH_4]^+$	43.4	20.0	99.01	238.2301	256.2645	2.0	↓ (0.93)

HexCer (Hexosylceramide); TG(Triacylglycerol), SPH (Sphingoid Base)
Fold Change = Medicated PD (Intensity) / Control (Intensity)

Table S5B. A list of the database matches based upon accurate mass (Lipid Maps and METLIN, 10 ppm) for VIP compounds found only in drug naïve PD *vs.* control analysis (VIP > 1).

Measured Feature	Database Matches	Formula	Adduct	Neutral Mass	Δ ppm	Expression Medicated PD (Fold Change)	Lipid Maps	METLIN
m/z 664.6213	Acer(42:1);O2	$C_{42}H_{81}NO_4$	$[M+H]^+$	663.6160	3.8	↓ (0.46)	✓	
	HexCer(36:1);O*						✓	
	Cer(42:2);O3*						✓	
	ACer(42:0)O3	$C_{42}H_{83}NO_5$	$[M-H_2O+H]^+$	681.6265	3.8		✓	
	Cer(42:1);O4*						✓	
	DG O(39:3)	$C_{42}H_{78}O_4$	$[M+NH_4]^+$	646.5894	3.8		✓	
m/z 641.4795	CAR(28:4);O4	$C_{35}H_{61}NO_8$	$[M+NH_4]^+$	623.4391	9.4	↑ (1.07)	✓	
	HexCer(29:4);O2						✓	
	ST 27:0;HexNAc						✓	
	LPG O(27:0);O	$C_{33}H_{69}O_9P$	$[M+H]^+$	640.4657	6.7		✓	
	CE(11:0);O4	$C_{38}H_{66}O_6$	$[M+Na]^+$	618.4837	6.7		✓	
	DG(35:4);O						✓	
	DG O(35:5);O2						✓	
	TG(35:3)						✓	
	TG O(35:4);O						✓	
	NAT(30:0);O4	$C_{32}H_{65}NO_8S$	$[M+NH_4]^+$	623.4391	4.1		✓	
	CE(13:3);O4	$C_{40}H_{64}O_6$	$[M+H]^+$	640.4657	3.0		✓	
	DG(37:7);O						✓	
	DG O(37:8);O2						✓	
	TG(37:6)						✓	
	TG O(37:7);O						✓	
	DG(37:6);O2	$C_{40}H_{66}O_7$	$[M-H_2O+H]^+$	658.4762	3.0		✓	

	TG(37:5);O						✓	
	TG O(37:6);O2						✓	
m/z 553.4300	NAT(26:0);O2	$C_{28}H_{57}NO_6S$	$[M+NH_4]^+$	535.3901	9.9	↑ (1.04)	✓	
	DG O(33:8)	$C_{36}H_{56}O_4$	$[M+H]^+$	552.4167	8.9		✓	
	DG(33:6)	$C_{36}H_{58}O_5$	$[M-H_2O+H]^+$	570.4272	8.9		✓	
	DG O(33:7);O						✓	
	MG(33:7);O						✓	
	TG O(33:6)						✓	
	CerP(28:0);O2	$C_{28}H_{58}NO_6P$	$[M+NH_4]^+$	535.3901	7.2		✓	
	LPC O(20:1)*						✓	
	LPE O(23:1)						✓	
	PC(20:0)						✓	✓
	PC(20:1)							✓
m/z 402.3932	FA(24:0);O*	$C_{24}H_{48}O_3$	$[M+NH_4]^+$	535.3901	2.5	↓ (0.54)	✓	✓
	MG O(21:1)						✓	
m/z 338.3426	NAE(20:0)*	$C_{22}H_{45}NO_2$	$[M-H_2O+H]^+$	570.4272	2.7	↓ (0.88)	✓	✓
	Docosenamide	$C_{22}H_{43}NO$	$[M+H]^+$	337.3345	2.5			✓
	Cyclohexanecarbon-ylpentadecylamine							✓
m/z 369.3836	-	-	-	-	-	↓ (1.02)		
m/z 340.3935	-	-	-	-	-	↓ (0.11)		

ACer (O-Acyl Ceramide); Cer (Ceramide); DG O (Alkylacylglycerol); CAR (Acyl Carnitine); HexCer (Glucosylceramide); ST;HexNAc (Sterol N-Acetyl-Hexosamine conjugates); LPG O (Monoalkylglycerophosphoglycerol); CE (Cholesterol Esters); DG (Diacylglycerol); TG (Triaylglycerol); TG O (alkyldiacylglyerol); NAT (N-Acyl Taurine); MG (Monoacylglycerol); CerP (Ceramide Phosphate); LPC O (Monoalkylglycerophosphocholine); LPE O (Monoalkylglycerophosphoethanolamine); NAE (N-Acyl Ethanolamine)

The number of oxygen atoms that are not included in the class-specific functional group is added after the semi-colon, where applicable

Lipid ID * corresponds to a Lipid Maps Structure Database (LMSD) hit which comprises only biologically relevant lipids to mammalian species. All other database matches correspond to Lipid Maps Computationally-generated species (COMP_DB) and METLIN as indicated in the final two columns

Fold Change = Medicated PD (Intensity) / Control (Intensity)

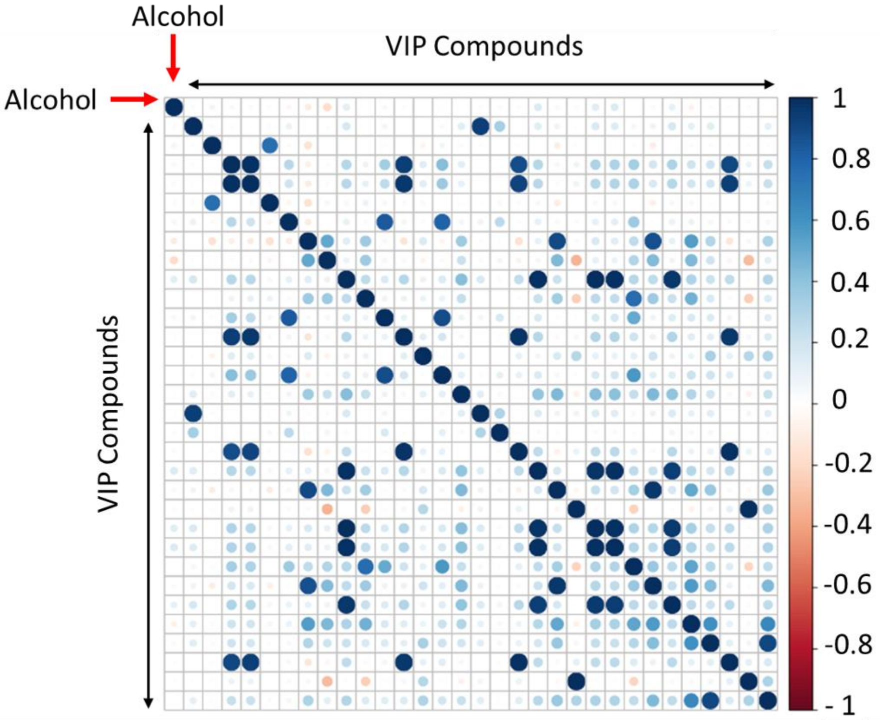

Figure S3. Pearson's correlation matrix displaying the relationship between the number of alcohol units consumed per participant and the intensity of each corresponding VIP compound from PLS-DA modelling. The size of each circle corresponds to the *p*-value and the colour relates to Pearson's *R* value, a strong correlation would yield either a 1 (deep blue) or -1 (deep red) association. The plot displays that there are relationships between VIP compounds but not between any specific compound and alcohol intake. This is highlighted by the lack of circles in the first row or column which both correspond to alcohol.

Table S6. Mummichog output from MetaboAnalyst analysis for pathways with $p < 0.05$ in drug naïve PD *vs.* control cohorts

Pathway	Pathway Size (*n*=)	Metabolite Hits (*n*=)	Significant Metabolite Hits (*n*=)	*p*-value	gamma-*p*	Enrichment Factor
Carnitine shuttle	72	26	14	0.002	0.007	1.69
Valine, leucine and isoleucine degradation	40	15	11	0.003	0.014	1.19
Fatty acid biosynthesis	49	7	6	0.010	0.061	0.53
Sphingolipid metabolism	25	7	6	0.010	0.061	1.04
Arachidonic acid metabolism	62	11	8	0.014	0.056	0.74
Primary bile acid biosynthesis	47	8	6	0.028	0.115	0.52
Fatty acid metabolism	50	10	7	0.029	0.104	1.12
Ether lipid metabolism	23	2	2	0.038	0.351	0.69
Vitamin E metabolism	54	37	23	0.038	0.075	2.08

Table S7. Mummichog output from MetaboAnalyst analysis for pathways with $p < 0.05$ in medicated PD *vs.* control cohorts

Pathway	Pathway Size (*n*=)	Metabolite Hits (*n*=)	Significant Metabolite Hits (*n*=)	*p*-value	gamma-*p*	Enrichment Factor
Steroid hormone biosynthesis	99	29	26	5.02×10^{-9}	5.64×10^{-8}	1.09
Carnitine shuttle	72	26	12	5.09×10^{-5}	2.04×10^{-3}	2.21
Arachidonic acid metabolism	62	11	8	3.09×10^{-4}	2.44×10^{-3}	0.96
Retinol metabolism	22	8	6	0.003	0.133	1.13
Sphingolipid metabolism	25	7	4	0.017	0.093	1.50
Prostaglandin formation from dihomo-γ-linoleic acid	11	5	3	0.038	0.205	3.61

Table S8. Details of the collecting sites in the UK/NL and the lead PI at each site

Site number	Site name	PI
1	Addenbrookes (Cambridge)	Paul Worth
2	Bournemouth	Khaled Amar
3	Cornwall/Truro	Christine Schofield
4	Lothian - Western General Edinburgh	Gordon Duncan
5	Edinburgh – Royal Infirmary of Edinburgh	Gordon Duncan
6	Edinburgh - Primary Care NHS Lothian (Seb Derm)	Richard Weller
7	Hampshire	Sam Arianayagam
8	Nottingham	Gill Sare
9	Pennine	Jason Raw
10	Salford	Monty Silverdale
11	Salisbury	Diran Padiachy
12	Sheffield	Oliver Bandmann
13	South Tees	Neil Archibold
14	Southern Health	Helen Roberts
15	Luton & Dunstable	Anette Schrag
16	Portsmouth	Sean Slaght
17	Northumbria	Richard Walker
18	London North West	Sophie Molloy
19	Bath	Veronica Lyell
20	Gateshead	Richard Athey
21	Sunderland	Uma Nath
22	Plymouth	Camille Caroll
23	Newcastle Upon Tyne Hospitals NHS Foundation Trust (Newcastle University)	Nicola Pavese
24	Royal Devon and Exeter NHS Foundation Trust	Robert James
25	Imperial College Healthcare NHS Trust	Sophie Molloy
26	LEAP Trial, The Netherlands	Sven Suwijn
