# Supplementary material for: Metabolomics of sebum reveals lipid dysregulation in Parkinson’s disease
Source: Nat Commun. 2021 Mar 11;12:1592. doi: 10.1038/s41467-021-21669-4 (PMC7952564; doi:10.1038/s41467-021-21669-4)
Supplement: Supplementary file 3 — Reporting Summary [file 41467_2021_21669_MOESM3_ESM.pdf]

nature research

Corresponding author(s): Prof. Perdita Barran

Last updated by author(s): Oct 16, 2020

Reporting Summary

Nature Research wishes to improve the reproducibility of the work that we publish. This form provides structure for consistency and transparency in reporting. For further information on Nature Research policies, seeAuthors & Referees and theEditorial Policy Checklist .

Statistics

For all statistical analyses, confirm that the following items are present in the figure legend, table legend, main text, or Methods section.

n/a	Confirmed	
☐	☒	The exact sample size (*n*) for each experimental group/condition, given as a discrete number and unit of measurement
☐	☒	A statement on whether measurements were taken from distinct samples or whether the same sample was measured repeatedly
☐	☒	The statistical test(s) used AND whether they are one- or two-sided<br>*Only common tests should be described solely by name; describe more complex techniques in the Methods section.*
☐	☒	A description of all covariates tested
☐	☒	A description of any assumptions or corrections, such as tests of normality and adjustment for multiple comparisons
☐	☒	A full description of the statistical parameters including central tendency (e.g. means) or other basic estimates (e.g. regression coefficient) AND variation (e.g. standard deviation) or associated estimates of uncertainty (e.g. confidence intervals)
☐	☒	For null hypothesis testing, the test statistic (e.g. *F*, *t*, *r*) with confidence intervals, effect sizes, degrees of freedom and *P* value noted<br>*Give P values as exact values whenever suitable.*
☒	☐	For Bayesian analysis, information on the choice of priors and Markov chain Monte Carlo settings
☒	☐	For hierarchical and complex designs, identification of the appropriate level for tests and full reporting of outcomes
☐	☒	Estimates of effect sizes (e.g. Cohen's *d*, Pearson's *r*), indicating how they were calculated

Our web collection on statistics for biologists contains articles on many of the points above.

Software and code

Policy information about availability of computer code

Data collection	MassLynx 4.2 (Waters Corp.) was used for LC -MS data acquisition
Data analysis	LC-MS raw data were deconvolved using Progenesis QI (Waters, Wilmslow, UK). Peak picking, alignment and area normalisation were carried out with reference to a pooled QC using default parameters. Features extracted from raw data were annotated using accurate mass match with METLIN (https://metlin.scripps.edu/), Human Metabolome Database (HMDB) and LipidMaps (https://www.lipidmaps.org/).<br>The data were mean centered and auto-scaled and missing values were replaced with cubic spline interpolation in MATLAB 2019a (MathWorks) prior to statistical analysis.<br>PLS-DA was performed for classification and prediction of data; re-sampling with replacement (bootstrapping) was used for model validation where the correct classification rates (CCRs) from the Y-variable were computed for the (n=250) test data sets. Algorithm used in the script that was used in MATLAB (2019a) to perform PLS-DA, is hosted at www.biospec.net (code available at https://github.com/Biospec/cluster-toolbox-v2.0/blob/master/cluster_toolbox/plsda_boots.m). Univariate ROC analysis was performed in Origin (Version 2017, OriginLab Corporation, Northampton, MA, USA) and multivariate ROC curve based exploratory analysis was executed using MetaboAnalyst Biomarker Analysis (Version 4.0) in which the data matrix was auto-scaled and PLS-DA was used for the classification method and feature ranking method with a two latent variable input.<br>LC-MSE raw data were deconvolved using Progenesis QI (Waters, Wilmslow, UK). Peak picking, alignment and area normalisation were carried out using one of the QC data files as the reference. Significant features extracted from raw data were aligned to significant features in clinical samples, using a RT window ±15 s and mass tolerance ±10 ppm filters. Features were annotated using accurate mass match and tandem MS data with Lipid Maps (https://www.lipidmaps.org/), Lipid Blast (https://fiehnlab.ucdavis.edu/projects/lipidblast and METLIN (https://metlin.scripps.edu/). Mass tolerances of 10 ppm and 30 ppm were applied for precursor and fragment ions, respectively. Compounds with a fragmentation score < 20 were not annotated. Progenesis QI score, fragmentation score and isotope similarity are reported for all annotations based on a combination of accurate mass and fragmentation data.

For manuscripts utilizing custom algorithms or software that are central to the research but not yet described in published literature, software must be made available to editors/reviewers. We strongly encourage code deposition in a community repository (e.g. GitHub). See the Nature Research guidelines for submitting code & software for further information.

Data

Policy information about availability of data

All manuscripts must include a data availability statement. This statement should provide the following information, where applicable:

- Accession codes, unique identifiers, or web links for publicly available datasets
- A list of figures that have associated raw data
- A description of any restrictions on data availability

Raw and processed data sets generated during and/or analysed during the current study are available from MetaboLights Repository, Study Identifier MTBLS2266. Source data are provided with this paper. Annotation of metabolites utilised publicly available databases such as LipidMaps (https://www.lipidmaps.org/), METLIN (https://metlin.scripps.edu/) and LipidBlast (https://fiehnlab.ucdavis.edu/projects/lipidblast) and HMDB (https://hmdb.ca).

Field-specific reporting

Please select the one below that is the best fit for your research. If you are not sure, read the appropriate sections before making your selection.

[x] Life sciences [ ] Behavioural & social sciences [ ] Ecological, evolutionary & environmental sciences

For a reference copy of the document with all sections, see nature.com/documents/nr-reporting-summary-flat.pdf

Life sciences study design

All studies must disclose on these points even when the disclosure is negative.

Sample size	A total of 274 participants were recruited from three subject groups: control (n=56), drug naïve PD (n=80), medicated PD (n=138). It is difficult to perform sample size calculations for untargeted metabolomics data due to the lack of pilot data and the highly multivariate nature of the data. It is generally accepted that over 100 samples within a study is a minimum sample size to derive sufficient statistical power. (Trivedi, D. K., Hollywood, K. A. & Goodacre, R. New Horizons in Translational Medicine Metabolomics for the masses: The future of metabolomics in a personalized world. New Horizons Transl. Med. 3, 294–305 (2017).)
Data exclusions	We excluded data from control participants who accompanied PD participants to their neurological appointment (paired control) since we found evidence of contamination in the sebum from some of these individuals. We also recruited another control cohort from a separate scheme (independent controls) who were not linked to a PD participant. The contamination hypothesis was tested using PLS-DA classification models between 'paired control' and 'independent control' cohorts which found high levels of classification between these control types. This is further supported by the poor classification seen between 'paired control' vs. both PD cohorts in comparison to the independent control vs. PD models which indicated an overlap in sebum signature within the paired control subjects.
Replication	Biological replicate samples were analysed from control (n=56), drug naïve PD (n=80), medicated PD (n=138) participants, The findings were not replicated in any independent cohort however, re-sampling methods were used to ensure the models are trained on a subset of data and tested on unseen data from the experiment.
Randomization	Samples were randomized (with stratification) such that each analytical batch had equal distribution of samples within groups. This was done to ensure in case of errors or instrument failure during analysis, we do not lose a large number of samples specific to any group. Subsequently, within each batch samples were randomized and also blinded (see below).
Blinding	During data collection, randomised batches of samples were blinded by a person in the group who was not collecting data. For example, 35 samples that were stratified in batch 1, were randomised and then labelled 1 to 35 and were revealed as samples 1 to 35 to the investigator during data collection. Batch 2 samples were randomised and then labelled 36 to 70 and so on. These blinding were removed once the data was collected in order to use supervised multivariate analysis that requires group labelling to be known to the statistician prior to analysis.

Reporting for specific materials, systems and methods

We require information from authors about some types of materials, experimental systems and methods used in many studies. Here, indicate whether each material, system or method listed is relevant to your study. If you are not sure if a list item applies to your research, read the appropriate section before selecting a response.

Materials & experimental systems

n/a	Involved in the study	
[x]	[ ]	Antibodies
[x]	[ ]	Eukaryotic cell lines
[x]	[ ]	Palaeontology
[x]	[ ]	Animals and other organisms
[ ]	[x]	Human research participants
[x]	[ ]	Clinical data

Methods

n/a	Involved in the study	
[x]	[ ]	ChIP-seq
[x]	[ ]	Flow cytometry
[x]	[ ]	MRI-based neuroimaging

Human research participants

Policy information about studies involving human research participants

Population characteristics	Population characteristics/demographics information has been summarised within the manuscript table 1. Key covariates for metabolomics analyses in human participants are: age, gender, BMI, smoking, drinking, diet and medication. For this analysis we collected age, gender, bmi, smoking, alcohol consumption and medication history of participants. We tested for these covariates using multivariate analysis and have reported these in our manuscript.
Recruitment	The participants included in this study were part of a nationwide recruitment process taking place at 25 different NHS clinics, in addition to subjects (n=4) that participated in a clinical trial in the Netherlands. All sites followed a standard sampling protocol provided to sites along with illustrations about sampling. Participants were offered to take part in research following a clinical diagnosis of PD and their partners were offered a choice to be a control participant in the research. We recruited controls who did not live with anyone with PD, separately at sites involved in dementia and ageing research, following the same sample protocol. Participants were sampled at four point on their top backs by applying stable pressure, using a medical gauze each time. The gauze samples (x2) were packed in inert plastic bags and sealed. They were sent back along with questionnaire that contained metadata. The samples were returned and stored within 24h from sampling, at -80 degrees celcius until ready for extraction.<br><br>The criteria for inclusion of a participant in the study was merely the diagnosis or lack of diagnosis of Parkinson's disease, for PD and control cohorts, respectively. Participants were recruited from a large geographical location from 28 NHS neurological clinics across the UK and four clinics in the Netherlands. Following recruitment of a large number of participants, samples were chosen to be included in this analysis based upon the metadata provided in their patient questionnaire. This was to ensure potential confounding factors could be controlled to the best of our ability between disease and control cohort groups. More men are diagnosed with PD than women which led to a large proportion of male recruitment, conversely more women had volunteered to be sampled as control participants leading to an imbalance in the gender of the disease-control cohorts. We evaluated the potential effect of gender imbalance within our data analysis and this is reported within the manuscript.
Ethics oversight	Ethical approval for this project (IRAS project ID 191917) was obtained by the NHS Health Research Authority (REC reference: 15/SW/0354). Informed consent was received from all participants prior to their enrolment in the study.

Note that full information on the approval of the study protocol must also be provided in the manuscript.
